# Determinants of the Use of Health and Fitness Mobile Apps by Patients With Asthma: Secondary Analysis of Observational Studies

**DOI:** 10.2196/25472

**Published:** 2021-09-22

**Authors:** Ana Luísa Neves, Cristina Jácome, Tiago Taveira-Gomes, Ana Margarida Pereira, Rute Almeida, Rita Amaral, Magna Alves-Correia, Sandra Mendes, Cláudia Chaves-Loureiro, Margarida Valério, Cristina Lopes, Joana Carvalho, Ana Mendes, Carmelita Ribeiro, Sara Prates, José Alberto Ferreira, Maria Fernanda Teixeira, Joana Branco, Marta Santalha, Maria João Vasconcelos, Carlos Lozoya, Natacha Santos, Francisca Cardia, Ana Sofia Moreira, Luís Taborda-Barata, Cláudia Sofia Pinto, Rosário Ferreira, Pedro Morais Silva, Tania Monteiro Ferreira, Raquel Câmara, Rui Lobo, Diana Bordalo, Cristina Guimarães, Maria Espírito Santo, José Ferraz de Oliveira, Maria José Cálix Augusto, Ricardo Gomes, Inês Vieira, Sofia da Silva, Maria Marques, João Cardoso, Ana Morete, Margarida Aroso, Ana Margarida Cruz, Carlos Nunes, Rita Câmara, Natalina Rodrigues, Carmo Abreu, Ana Luísa Albuquerque, Claúdia Vieira, Carlos Santos, Rosália Páscoa, Carla Chaves-Loureiro, Adelaide Alves, Ângela Neves, José Varanda Marques, Bruno Reis, Manuel Ferreira-Magalhães, João Almeida Fonseca

**Affiliations:** 1 Department of Community Medicine, Information and Health Decision Sciences (MEDCIDS) Faculty of Medicine University of Porto Porto Portugal; 2 Center for Health Technology and Services Research (CINTESIS) Faculty of Medicine University of Porto Porto Portugal; 3 Imperial NIHR Patient Safety Translational Research Centre Institute of Global Health Innovation Imperial College London London United Kingdom; 4 Instituto de Investigação e Formação Avançada em Ciências e Tecnologias da Saúde Institute of Research and Advanced Training in Health Sciences and Technologies, University Institute of Health Sciences Cooperativa de Ensino Superior Politécnico e Universitário, CRL Gandra Portugal; 5 Faculdade de Ciências da Saúde Universidade Fernando Pessoa Porto Portugal; 6 Allergy Unit Instituto and Hospital CUF Porto Portugal; 7 Department of Cardiovascular and Respiratory Sciences Porto Health School Polytechnic Institute of Porto Porto Portugal; 8 Department of Women’s and Children’s Health, Paediatric Research Uppsala University Uppsala Sweden; 9 Serviço Pneumologia Hospitais da Universidade de Coimbra Coimbra Portugal; 10 Unidade de Imunoalergologia Hospital Pedro Hispano Unidade Local de Saúde de Matosinhos Matosinhos Portugal; 11 Imunologia Básica e Clínica Faculdade de Medicina Universidade do Porto Porto Portugal; 12 Serviço de Pediatria Hospital Pedro Hispano Unidade Local de Saúde de Matosinhos Matosinhos Portugal; 13 Serviço de Imunoalergologia Hospital de Santa Maria Centro Hospitalar Lisboa Norte Lisboa Portugal; 14 Serviço de Imunoalergologia Centro Hospitalar e Universitário de Coimbra Coimbra Portugal; 15 Serviço de Imunoalergologia Hospital de Dona Estefânia Centro Hospitalar Universitário de Lisboa Central Lisboa Portugal; 16 Serviço de Imunoalergologia Unidade I, Centro Hospitalar Vila Nova de Gaia/Espinho Vila Nova de Gaia Portugal; 17 Serviço de Pediatria Centro Materno Infantil do Norte Centro Hospitalar Universitário do Porto Porto Portugal; 18 Serviço de Pneumologia Hospital Beatriz Ângelo Loures Portugal; 19 Serviço de Pediatria Hospital da Senhora da Oliveira Guimarães Portugal; 20 Serviço de Imunoalergologia Centro Hospitalar Universitário de São João Porto Portugal; 21 Serviço de Imunoalergologia Hospital Amato Lusitano Unidade Local de Saúde de Castelo Branco Castelo Branco Portugal; 22 Serviço de Imunoalergologia Centro Hospitalar Universitário do Algarve Portimão Portugal; 23 Unidade de Saúde Familiar Terras de Azurara Agrupamento de Centros de Saúde Dão Lafões Mangualde Portugal; 24 Unidade de Imunoalergologia Hospital do Divino Espirito Santo Ponta Delgada Portugal; 25 Department of Allergy & Clinical Immunology Cova da Beira University Hospital Centre Covilhã Portugal; 26 Centro de Investigação em Ciências da Saúde - Health Sciences Research Centre & NuESA –Environment & Health Study Group Faculty of Health Sciences University of Beira Interior Covilhã Portugal; 27 Serviço de Pneumologia Hospital São Pedro de Vila Real Centro Hospitalar de Trás-os-Montes e Alto Douro Vila Real Portugal; 28 Departamento de Pediatria Hospital de Santa Maria Centro Hospitalar de Lisboa Norte Lisboa Portugal; 29 Imunoalergologia Grupo HPA Saúde Portimão Portugal; 30 Unidade de Saúde Familiar Progresso e Saúde Agrupamento de Centros de Saúde Baixo Mondego Tocha Portugal; 31 Serviço de Pneumologia Hospital Nossa Senhora do Rosário Centro Hospitalar Barreiro Montijo Barreiro Portugal; 32 Unidade de Saúde Familiar João Semana Agrupamento de Centros de Saúde Baixo Vouga Ovar Portugal; 33 Serviço de Pediatria Unidade Hospitalar de Famalicão Centro Hospitalar do Médio Ave Vila Nova de Famalicão Portugal; 34 Unidade de Saúde Familiar Caminhos do Cértoma Agrupamento de Centros de Saúde Baixo Mondego Pampilhosa Portugal; 35 Unidade de Saúde Familiar Arte Nova Agrupamento de Centros de Saúde Baixo Vouga Oliveirinha Portugal; 36 Imunoalergologia Hospital Privado de Alfena Trofa Saúde Alfena Portugal; 37 Serviço de Pediatria Hospital de São Teotónio Centro Hospitalar Tondela–Viseu Viseu Portugal; 38 Serviço de Pneumologia Hospital Garcia de Orta Almada Portugal; 39 Unidade de Cuidados Saúde Personalizados Arnaldo Sampaio Agrupamento de Centros de Saúde Pinhal Litoral Leiria Portugal; 40 Unidade de Saúde Familiar Cuidarte Unidade Local de Saúde do Alto Minho Portuzelo Portugal; 41 Serviço de Imunoalergologia Centro Hospitalar Universitário do Porto Porto Portugal; 42 Serviço de Pneumologia Hospital Santa Marta Centro Hospitalar Universitário de Lisboa Central Lisboa Portugal; 43 Serviço de Imunoalergologia Hospital Infante D Pedro Centro Hospitalar Baixo Vouga Aveiro Portugal; 44 Unidade de Saúde Familiar Pedras Rubras Agrupamento de Centros de Saúde do Grande Porto III - Maia/Valongo Maia Portugal; 45 Unidade de Saúde Familiar Bom Porto Agrupamento de Centros de Saúde do Grande Porto V - Porto Ocidental Porto Portugal; 46 Imunoalergologia Centro de Imunoalergologia do Algarve Portimão Portugal; 47 Serviço de Imunoalergologia Serviço de Saúde da Região Autónoma da Madeira Funchal Portugal; 48 Unidade de Saúde Familiar Mondego Agrupamento de Centros de Saúde Baixo Mondego Coimbra Portugal; 49 Serviço de Imunoalergologia Hospital São Pedro de Vila Real Centro Hospitalar De Trás-Os-Montes E Alto Douro Vila Real Portugal; 50 Unidade de Saúde Familiar Coimbra Centro Agrupamento de Centros de Saúde Baixo Mondego Coimbra Portugal; 51 Unidade de Saúde Familiar Corgo Agrupamentos de Centros de Saúde Douro I - Marão e Douro Norte Vila Real Portugal; 52 Unidade de Saúde Familiar Santo António Agrupamento de Centros de Saúde do Cávado III - Barcelos/Esposende Barcelos Portugal; 53 Unidade de Saúde Familiar Abel Salazar Agrupamento de Centros de Saúde do Gaia Vila Nova de Gaia Portugal; 54 Serviço Pediatria Ambulatória Centro Hospitalar e Universitário de Coimbra Coimbra Portugal; 55 Serviço de Pneumologia Unidade I Centro Hospitalar Vila Nova de Gaia/Espinho Vila Nova de Gaia Portugal; 56 Unidade de Saúde Familiar Araceti Agrupamento de Centros de Saúde Baixo Mondego Arazede Portugal; 57 Unidade de Saúde Familiar Viseu-Cidade Agrupamento de Centros de Saúde do Dão Lafões Viseu Portugal; 58 Unidade de Cuidados Saúde Personalizados Sicó Agrupamento de Centros de Saúde Pinhal Litoral Leiria Portugal

**Keywords:** mobile apps, smartphone, patient participation, self-management, asthma

## Abstract

**Background:**

Health and fitness apps have potential benefits to improve self-management and disease control among patients with asthma. However, inconsistent use rates have been reported across studies, regions, and health systems. A better understanding of the characteristics of users and nonusers is critical to design solutions that are effectively integrated in patients’ daily lives, and to ensure that these equitably reach out to different groups of patients, thus improving rather than entrenching health inequities.

**Objective:**

This study aimed to evaluate the use of general health and fitness apps by patients with asthma and to identify determinants of usage.

**Methods:**

A secondary analysis of the INSPIRERS observational studies was conducted using data from face-to-face visits. Patients with a diagnosis of asthma were included between November 2017 and August 2020. Individual-level data were collected, including age, gender, marital status, educational level, health status, presence of anxiety and depression, postcode, socioeconomic level, digital literacy, use of health services, and use of health and fitness apps. Multivariate logistic regression was used to model the probability of being a health and fitness app user. Statistical analysis was performed in R.

**Results:**

A total of 526 patients attended a face-to-face visit in the 49 recruiting centers and 514 had complete data. Most participants were ≤40 years old (66.4%), had at least 10 years of education (57.4%), and were in the 3 higher quintiles of the socioeconomic deprivation index (70.1%). The majority reported an overall good health status (visual analogue scale [VAS] score>70 in 93.1%) and the prevalence of anxiety and depression was 34.3% and 11.9%, respectively. The proportion of participants who reported using health and fitness mobile apps was 41.1% (n=211). Multivariate models revealed that single individuals and those with more than 10 years of education are more likely to use health and fitness mobile apps (adjusted odds ratio [aOR] 2.22, 95%CI 1.05-4.75 and aOR 1.95, 95%CI 1.12-3.45, respectively). Higher digital literacy scores were also associated with higher odds of being a user of health and fitness apps, with participants in the second, third, and fourth quartiles reporting aORs of 6.74 (95%CI 2.90-17.40), 10.30 (95%CI 4.28-27.56), and 11.52 (95%CI 4.78-30.87), respectively. Participants with depression symptoms had lower odds of using health and fitness apps (aOR 0.32, 95%CI 0.12-0.83).

**Conclusions:**

A better understanding of the barriers and enhancers of app use among patients with lower education, lower digital literacy, or depressive symptoms is key to design tailored interventions to ensure a sustained and equitable use of these technologies. Future studies should also assess users’ general health-seeking behavior and their interest and concerns specifically about digital tools. These factors may impact both initial engagement and sustained use.

## Introduction

Smart mobile technology has revolutionized how we communicate, share, and consume content, seeping into many different sectors of society, including health care [1]. With the democratization of smartphone use, with 3.8 billion smartphone users worldwide [2], the market of specific apps has experienced a boom. Often free, easy to download, and easy to use, mobile apps have an extensive application in social, educational, and entertainment fields and naturally in the fields of self-management and health behavior change [3]. According to the software application industry, around 500 million smartphone users worldwide were using a health and fitness app in 2015; and by 2018, an estimated 50% of the 3.4 billion smartphone and tablet users, including health care professionals, consumers, and patients, would have downloaded one [4]. The total global mHealth market is predicted to reach the US $100 billion mark in 2021, which constitutes a 5-fold increase from 2016 [5]. In this context, it is hypothesized that apps may become ubiquitous solutions impacting a large number of patients, often capitalizing on gamification strategies and social interaction [6]. In particular, health and fitness apps are a promising approach for improving self-management behaviors in patients with asthma, a prevalent long-term condition with potential social and economic impacts [7,8], which requires a range of self-management skills in everyday life [9]. Indeed, around 1500 mobile apps are targeting patients with asthma in both the Apple App Store and the Google Play Store [10]. A systematic review published by Unni et al [11] suggests that the use of mobile apps by patients with asthma may have benefits across a range of outcomes, including medication adherence and asthma control.

However, the current use of smart devices and apps among patients with asthma remains unexplored, as emphasized by a position paper of the European Academy of Allergy and Clinical Immunology, highlighting the lack of published studies on the use of mHealth in allergic diseases [12]. While there are more than 100 papers published over the last 5 years, they either evaluate the characteristics of specific apps (rather than their use) or focus on the impact of asthma-specific apps.

A recent study reported that smart device ownership levels in patients with asthma are similar to those of the general population, that three-quarters of patients had downloaded/used a general app, yet only one-third had ever used a health and fitness app [13]. A significant variability exists in usage among different racial/ethnic and sociodemographic groups. Nonetheless, this evidence comes mainly from studies conducted in the United States and does not address specific disease contexts [14-18]. The use of health and fitness apps in the asthma context can be explored through the lens of the conceptual model developed by Andersen et al [19], which proposes that the use of health services is driven by three dynamics: predisposing factors (eg, age and gender), enabling factors (eg, socioeconomic level, education, and literacy), and need (eg, clinical characteristics and severity of disease).

The purpose of this study is to evaluate the use of health and fitness apps by patients with asthma and to identify determinants of usage. Specifically, we will investigate the following: (1) the proportion of patients with asthma using health and fitness apps and (2) the relationships among predisposing, enabling and need factors, and using mHealth apps.

## Methods

### Study Design

**Figure 1:**
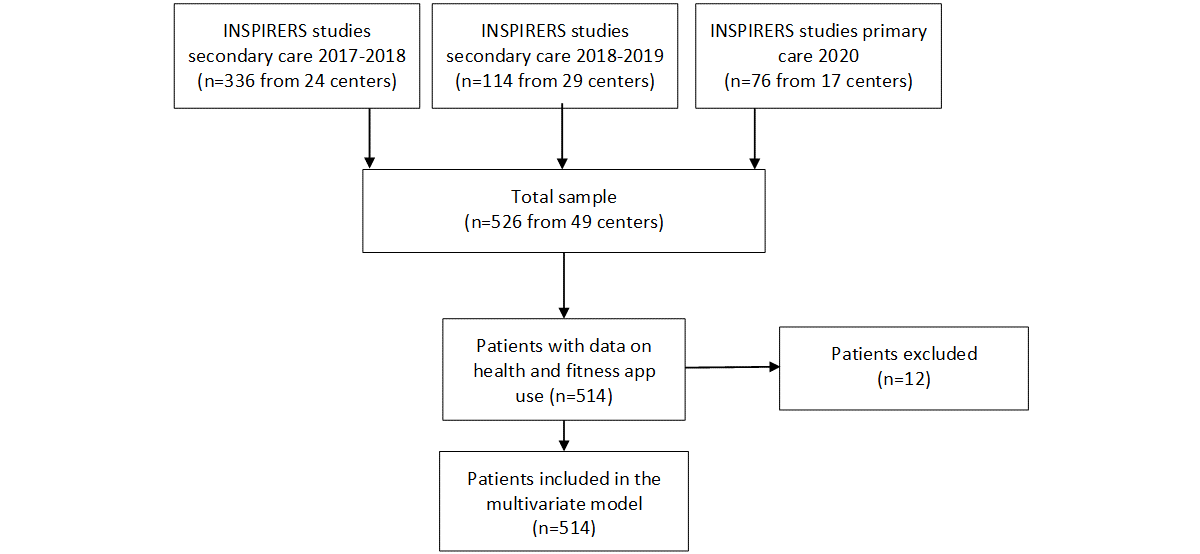
INSPIRERS studies flowchart.

A secondary analysis of INSPIRERS observational studies involving 32 secondary care centers (allergy, pulmonology, and pediatrics departments) and 17 primary care centers in Portugal was performed (Figure 1), as part of the INSPIRERS project. The design of the INSPIRERS observational studies was disseminated through email contacts, social networks, and oral communications at national meetings/conferences, and physicians/centers interested in being part of the study contacted the research team. A convenience sample of adolescents and adults with persistent asthma was recruited for the INSPIRERS studies between November 2017 and August 2020. Depending on the study, each center was asked to recruit a minimum of 2-10 patients. The 3 INSPIRERS observational studies address the topic of adherence to asthma inhalers among adolescents and adults with persistent asthma (Figure 1). Further details on the project setting and methods have been previously published [20,21]. This study is reported in accordance with the STROBE (Strengthening the Reporting of Observational Studies in Epidemiology) guidelines [22].

### Participants and Data Collection

Data were collected using a questionnaire during a face-to-face visit. The questionnaire had a section to be completed by the physician addressing patients’ asthma characteristics and a set of questions to be self-completed by the patient, as detailed below. Patients were included if they had a previous medical diagnosis of persistent asthma, were at least 13 years old, and had an active prescription for an inhaled controller medication for asthma. Patients were excluded if they had a diagnosis of a chronic lung disease other than asthma or a diagnosis of another significant chronic condition with possible interference with the study aims.

**Figure 2:**
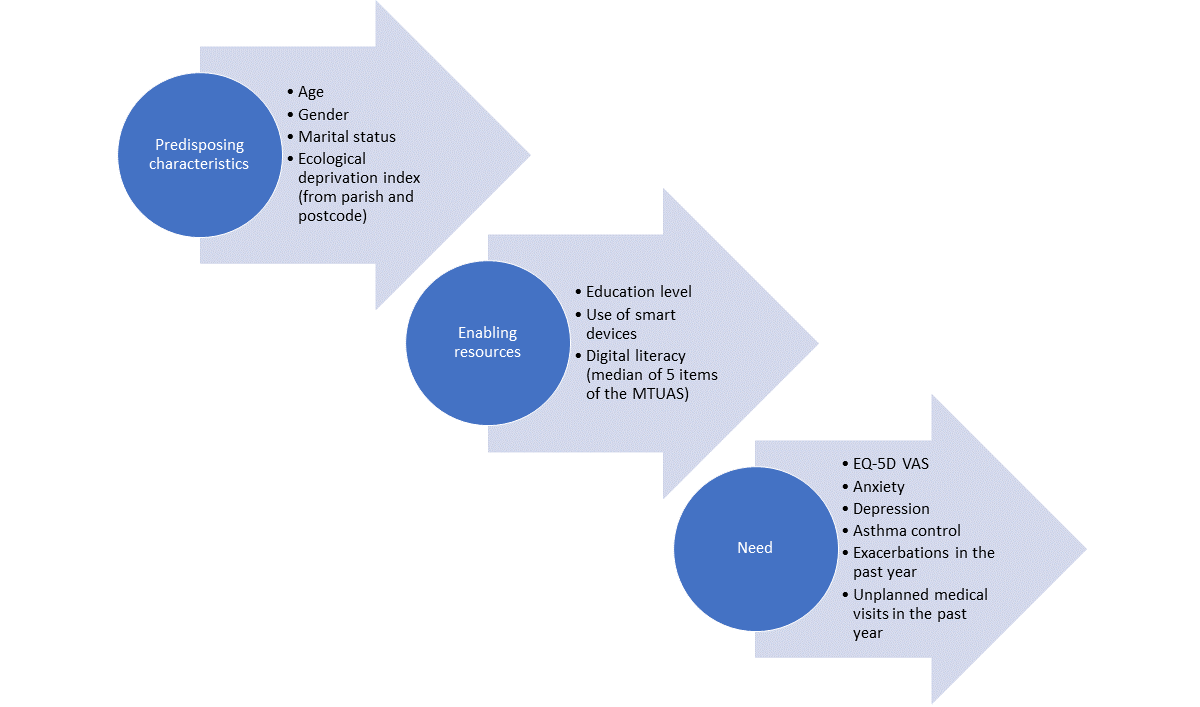
Variables collected included predisposing factors, enabling factors, and need according to the Andersen et al [19]. MTUAS: Media and Technology Usage and Attitudes Scale; VAS: Visual Analog Scale.

Users and nonusers were defined as individuals who answered “yes” or “no,” respectively, to the question “Have you ever downloaded and used a health and fitness app?” Health and fitness apps were defined as a range of apps related to personal fitness, workout tracking, diet and nutritional tips, health and safety, etc. In accordance with the conceptual model proposed by Andersen et al [19], variables collected included predisposing factors, enabling factors, and need (Figure 2). Predisposing factors included demographic data (ie, age, gender, marital status, parish, and postcode), and enabling factors included education level, use of smart devices, and digital literacy, both collected from patients. Digital literacy was defined as the median of 5 items of the Media and Technology Usage and Attitudes Scale (MTUAS, ie, use of the GPS, browsing the web, taking pictures, gaming, and checking social networks) rated by frequency of use in a 10-point Likert scale (1=never to 10=all the time) [23]. Additionally, socioeconomic level was explored as an enabling factor, which was defined as the Portuguese ecological deprivation index, extracted from the patient residence information (civil parish/postcode), and categorized into 5 quintiles (Q1=least deprived to Q5=most deprived) [24]. Need variables included smoking status, patients’ perceived overall health status (from EQ-5D Visual Analog Scale [VAS], ranging from 0 [worst imaginable health state] to 100 [best imaginable health state]) [25], the presence of anxiety or depression (cut-off≥8 in the Hospital Anxiety and Depression Subscales) [26], and physicians’ input, including asthma control level (uncontrolled, partially controlled, or well controlled according to the classification of the Global Initiative for Asthma [27]), number of exacerbations (episodes of progressive increase in shortness of breath, cough, wheezing, or chest tightness, requiring a change in maintenance therapy) in the past year [28], and the number of unplanned appointments in the past year.

Age was categorized into age bands (13-18,18-30, 30-40, 40-50, 50-65, and ≥65 years). Other continuous variables (socioeconomic level, median digital literacy, and overall health status) were categorized into quartiles.

### Data Analysis

Counts and proportions were calculated for each variable. Multivariate logistic regression with the Enter method was used to ascertain the determinants of being a user of a health and fitness mobile app (dependent variable) in accordance with Andersen et al’s [19] conceptual model. Categorical variables, such as gender, age, marital status, educational level, socioeconomic level, use of smart devices, digital literacy, overall health status, smoking status, presence of anxiety or depression, and asthma status were explored as independent variables, irrespective of significance in preliminary univariate logistic regressions. We assessed model fit using the pseudo-R2 Nagelkerke method and tested for evidence for poor model fit using the Hosmer–Lemeshow test. In addition, to assess the increasing contribution of each covariate to the model, we adopted a stepwise approach to build models starting from 1 covariate until including all covariates in the full models. For each model, we computed the described goodness of fit statistics. The quality of the final model was also assessed using the Aikake Information Criterion. Adjusted odds ratios and 95% CIs were calculated. Statistical analyses were conducted using R and the “glm” package. The map of Portugal was created using Paintmaps [29].

### Ethics Approval

The studies were approved by the ethics committees of all participating centers. The studies were conducted in accordance with the ethical standards established in the Declaration of Helsinki. Eligible patients were approached by physicians during medical visits and invited to participate. Written informed consent was obtained before enrollment. Adult patients signed a consent form; adolescents signed an assent form and a parental consent form was also obtained.

## Results

**Figure 3:**
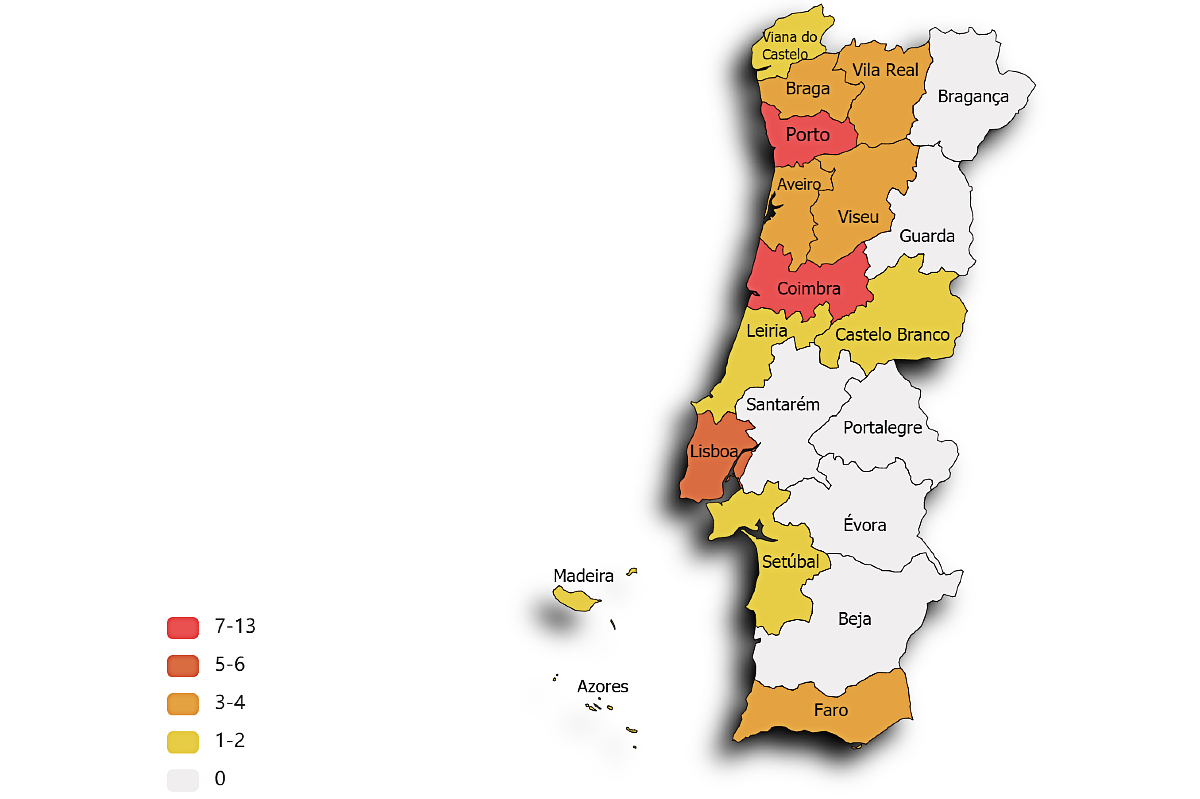
Number of participating centres per district.

A total of 526 patients attended a face-to-face visit between November 2017 and August 2020 at the 49 recruiting centers. Of those, 12 did not answer the question “Have you ever downloaded and used a health and fitness app?” and were excluded (Figure 1). The recruiting centers included 12 of the 18 Portuguese districts, which represented 9,189,723 inhabitants (89% of the total national population) [30]. A detailed overview of the distribution of the participating centers by district is provided in Figure 3.

The majority of the subjects were ≤40 years old (66.4%, n=341) and 63.4% (n=326) were female. Most participants were single (58.0%, n=298) and had at least 10 years of education (57.4%, n=295). Approximately one-third were in the 2 lower quintiles of the socioeconomic deprivation index (29.9%, n=154). Regarding general health status, as assessed by EQ-5D VAS, 69.8% (n=359) of the participants reported a score of ≥70. Most of the subjects were never smokers (75.5%, n=388). The prevalence of anxiety and depression symptoms in the sample was, respectively, 34.4% (n=177) and 11.9% (n=61).

Asthma was well controlled among 51.0% (n=262) of participants. While 50.8% (n=261) of participants had 1 or more asthma exacerbations during the last year, most of the participants did not have any unplanned appointments (66.1%, n=340) or inpatient admissions (94.2%, n=484). The proportion of participants who reported using health and fitness mobile apps was 41.1% (n=211). A full description of the sample, as well as the characteristics of the nonuser and user groups, is provided in Table 1.

Characteristics of both users and nonusers were explored using multivariate logistic regression. The aORs show that single individuals and those with more than 10 years of education are more likely to use health and fitness mobile apps (aOR 2.22, 95%CI 1.05-4.75, and aOR 1.95 95%CI 1.12-3.45, respectively). Higher digital literacy scores were also associated with higher odds of being a user of health and fitness apps, with participants in the second, third, and fourth quartiles showing, respectively, aORs of 6.74 (95%CI 2.90-17.40), 10.30 (95%CI 4.28-27.56), and 11.52 (95%CI 4.78-30.87). Participants with depression symptoms had lower odds of using health and fitness apps (aOR 0.32, 95%CI 0.12-0.83). No significant associations were found with gender, age, socioeconomic level, general health status, smoking status, anxiety, and asthma control (including level of control, number of inpatient admissions, or number of exacerbations). A detailed overview of the multivariate analysis is provided in Table 2.

**Table 1:** Characteristics of the participants according to their use of health and fitness mobile apps (N=514).

Characteristics			Nonusers (n=303), n (%)	Users (n=211), n (%)	Total, n (%)
**Sociodemographic**					
	**Gender**				
		Female	189 (62.4)	137 (64.9)	326 (63.4)
		Male	114 (37.6)	74 (35.1)	188 (36.6)
	**Age band (years)^a^**				
		13-18	92 (30.4)	62 (29.4)	154 (30.0)
		18-30	41 (13.5)	74 (35.1)	115 (22.4)
		30-40	40 (13.2)	32 (15.2)	72 (14.0)
		40-50	51 (16.8)	31 (14.7)	82 (16.0)
		50-65	51 (16.8)	7 (3.3)	58 (11.3)
		≥65	23 (7.6)	3 (1.4)	26 (5.1)
	**Marital status^b^**				
		Married	128 (42.2)	49 (23.2)	177 (34.4)
		Separated	19 (6.3)	11 (5.2)	30 (5.8)
		Single	149 (49.2)	149 (70.6)	298 (58.0)
		Widow	6 (2.0)	2 (1.0)	8 (1.6)
	**Education level (years)**				
		0-10	159 (52.5)	60 (28.4)	219 (42.6)
		>10	144 (47.5)	151 (71.6)	295 (57.4)
	**Socioeconomic level^c^**				
		Q1 (least deprived)	32 (10.6)	19 (9.0)	51 (9.9)
		Q2	55 (18.2)	48 (22.7)	103 (20.0)
		Q3	72 (23.8)	37 (17.5)	109 (21.2)
		Q4	72 (23.8)	61 (28.9)	133 (25.9)
		Q5 (most deprived)	64 (21.1)	40 (19.0)	104 (20.2)
**Digital use and literacy**					
	Use of smart devices		262 (86.5)	211 (100)	473 (92.0)
	**Mean digital literacy^d^**				
		Q1 (0-4.17)	82 (27.1)	10 (4.7)	92 (17.9)
		Q2 (4.17-5.67)	76 (25.1)	66 (31.3)	142 (28.6)
		Q3 (5.67-6.83)	51 (16.8)	66 (31.3)	117 (22.8)
		Q4 (6.83-10.00)	54 (17.8)	69 (32.7)	123 (23.9)
**General health status**					
	**Overall health^e^**				
		Q1 (0-70)	92 (30.4)	54 (25.6)	146 (28.4)
		Q2 (70-80)	68 (22.4)	53 (25.1)	121 (23.5)
		Q3 (80-90)	87 (28.7)	64 (30.3)	151 (29.4)
		Q4 (90-100)	49 (16.2)	38 (18.0)	87 (16.9)
	**Smoking status^b^**				
		Never smokers	237 (78.2)	151 (71.6)	388 (75.5)
		Former smokers	40 (13.2)	46 (21.8)	86 (16.7)
		Current smokers	25 (8.3)	14 (6.6)	39 (7.6)
	Anxiety symptoms^f^		110 (36.3)	67 (31.8)	177 (34.4)
	Depression symptoms^f^		52 (17.2)	9 (4.3)	61 (11.9)
**Asthma status**					
	**Asthma control^g^**				
		Well-controlled	152 (50.2)	110 (52.1)	262 (51.0)
		Partially/uncontrolled	150 (49.5)	98 (46.4)	248 (48.2)
	≥1 asthma exacerbation in the past year^h^		160 (52.8)	101 (47.9)	261 (50.8)
	≥1 unplanned appointment in the past year^c^		105 (34.7)	55 (26.1)	160 (31.1)
	≥1 inpatient admission in the past year^c^		11 (3.6)	5 (2.4)	16 (3.1)

^a^7 patients with missing data.

^b^1 patient with missing data.

^c^14 patients with missing data.

^d^40 patients with missing data.

^e^9 patients with missing data.

^f^2 patients with missing data.

^g^4 patients with missing data.

^h^15 patients with missing data.

**Table 2:** Multivariate analysis to explain the use of health and fitness apps.

Predictor		Odds ratio (5%-95% CI)	*P* value
**Gender**			
	Male	Reference^a^	
	Female	1.37 (0.86-2.20)	.19
**Age band (years)**			
	13-18	Reference	
	18-30	1.93 (0.97-3.89)	.06
	30-40	1.53 (0.59-4.05)	.38
	40-50	1.41 (0.53-3.77)	.49
	50-65	0.63 (0.12-2.86)	.57
	≥65	1.68 (0.19-11.47)	.62
**Marital status**			
	Married	Reference	
	Separated	1.64 (0.55-4.96)	.37
	Single	2.22 (1.05-4.75)	*.04^b^*
	Widow	1.17 (0.03-33.55)	.93
**Education level (years)**			
	0-10	Reference	
	>10	1.95 (1.12-3.45)	*.02*
**Socioeconomic level**			
	Q1 (least deprived)	Reference	
	Q2	2.02 (0.84-4.92)	.12
	Q3	1.08 (0.45-2.64)	.86
	Q4	1.42 (0.61-3.33)	.41
	Q5 (most deprived)	1.34 (0.56-3.24)	.52
**Mean digital literacy**			
	Q1 (0-4.17)	Reference	
	Q2 (4.17-5.67)	6.74 (2.90-17.40)	*<.001*
	Q3 (5.67-6.83)	10.30 (4.28-27.56)	*<.001*
	Q4 (6.83-10)	11.52 (4.78-30.87)	*<.001*
**Overall health**			
	Q1 (0-70)	Reference	
	Q2 (70-80)	1.05 (0.56-1.99)	.87
	Q3 (80-90)	0.87 (0.46-1.63)	.67
	Q4 (90-100)	0.86 (0.42-1.76)	.69
**Smoking status**			
	Never smokers	Reference	
	Former smokers	1.83 (0.97-3.53)	.07
	Current smokers	0.84 (0.34-2.05)	.70
**Anxiety symptoms**			
	No	Reference	
	Yes	1.12 (0.64-1.95)	.69
**Depression symptoms**			
	No	Reference	
	Yes	0.32 (0.12-0.83)	*.02*
**Asthma control**			
	Well-controlled	Reference	
	Partially/uncontrolled	0.89 (0.54-1.44)	.62
**Asthma exacerbation in the past year**			
	0	Reference	
	≥1	0.99 (0.56-1.75)	.96
**Unplanned appointment in the past year**			
	0	Reference	
	≥1	1.01 (0.52-1.91)	.99
**Inpatient admission in the past year**			
	0	Reference	
	≥1	1.73 (0.37-8.03)	.48

^a^Confidence intervals could not be calculated. Reference means the category used as reference (ie, to which other categories are compared). The Hosmer–Lemeshow Test yielded a *P* value of .21, *χ*^2^_8_=10.9, Aikake Information Criterion of 542, and coefficient of determination (*R*^2^) of 48%.

^b^Italicized *P* values are significant.

## Discussion

### Principal Findings

Use of health and fitness mobile apps was positively associated with a single status, >10 years of education, and higher digital literacy scores, and negatively associated with depressive symptoms. No significant associations were found with other variables, including gender, age, socioeconomic level, general health status, smoking status, anxiety, and asthma control.

### Comparison With Previous Studies

According to our results, single participants are more likely to use health and fitness apps. In a recent mixed methods study, Zhou et al [18] explored the barriers to and facilitators of the use of mobile health apps and found that single users had less strong concerns about information security and privacy and less desire to have stringent security protection, which could contribute to higher usage levels in this group.

Consistent with our findings, previous studies have described educational attainment as an important predictor of use of mobile devices and apps [14-16]. However, the relationship may be more complex than initially predicted, as in another study by Carroll et al [14] where both patients with a degree and those with less than high school education were significantly associated with a reduced likelihood of using health apps. The reasons for the educational differences are not fully understood but may reflect the effect of digital skills and confidence, and social norms related to the perceived value of using health and fitness apps [16].

A significant association between digital health literacy and use of health and fitness apps was also found. Digital health literacy has been previously shown to affect the use of health apps [17]. However, comparisons between different studies are limited by the heterogeneity of tools used to evaluate patients’ digital health literacy; therefore, standardization of the assessment methods used is recommended in the future. It is also important to note that although individuals with low general health literacy tend to use less health information technology [31], previous evidence has shown that tailored approaches including apps programmed with computer-animated characters, text, and graphics to provide health communication and education could be a widely accepted option for these patients [32].

Interestingly, our study found a negative association between the use of health and fitness apps and the presence of depressive symptoms. Despite the breadth of research exploring the determinants of use of mental health apps [33-35], there was a lack of evidence specifically exploring the impact of the presence of mental health symptoms on usage rates. However, several studies show that patients with depressive symptoms often exhibit poor engagement with health services, and health care avoidance [36,37], and such behaviors may also contribute to a lower interest and usage of health and fitness apps by these groups of patients. This finding opens a new research avenue, and future work should explore the drivers for this effect and should involve patients with mental health problems in the co-design of digital solutions and interventions that promote both early and sustained use. The other needed variables, such as the ones related to asthma control, were not significant in explaining mHealth behaviors, although they showed a similar trend to the one observed with the variable depression. This is in line with previous asthma mHealth studies showing that patients with worse asthma control engage less with the tested apps [15,38]. Regarding age, odds ratios were higher for patients aged 18-30 years (1.93, 95%CI 0.97-3.89), but no significant differences were detected, despite the *P* value being close to the significance threshold (*P*=.06). Increasing the sample size in future studies could help further explore this effect and to confirm or exclude a potential type II error [39]. Previous studies found that younger adults were more likely to engage with health apps [14-16] and suggested that the effect of age likely reflects both social norms and cohort effects, such as the increased exposure to these devices and apps at younger ages [14]. Previous evidence also suggests that younger adults seem to have higher digital health literacy levels, which can contribute to increased use of digital solutions [40]. While it is recognized that the use of digital health solutions by older persons could improve patient engagement and reduce both financial burden and pressure on health systems, usage rates among this group remain low. According to a mixed methods study conducted by Fox et al, this digital health divide is deepening owing to older adults’ perceived inability and unwillingness to use digital technologies, stemming from mistrust, high-risk perceptions, and a strong desire for privacy [41].

Finally, no significant associations were found with gender. Previous literature shows mixed evidence on this subject: while some studies had found a higher use among male subjects [15], others reported the opposite [14,16]. The reasons for gender differences in some samples are unclear but may reflect sample-specific differences in health-seeking behavior, and interest and participation in healthy lifestyle interventions in general.

### Strengths and Limitations

This study has several strengths. It is the first national-level study performed in Portugal, evaluating the use of health and fitness apps among patients with asthma, covering the majority of the geographic regions of the country. A comprehensive set of individual-level characteristics was collected and analyzed, which allowed us to explore the impact of a range of sociodemographic factors, health literacy, and cofactors such as general health status and asthma status. Although no power calculation was performed (which was associated with the secondary analysis nature of this study), the overall large sample size contributes to the robustness of these findings.

Other limitations also need to be acknowledged. Intrinsic to the study design using convenience sampling, a potential selection bias cannot be excluded. This can possibly explain the low number of patients above 65 years of age. Nevertheless, this risk was mitigated by sampling patients from different health care settings, centers, and geographic regions. Health and fitness app use was patient-reported; therefore, a potential information bias cannot be excluded either. As an alternative, future studies could use patient log-in as a measure of app use. Furthermore, digital literacy was assessed using selected items of MTUAS scale, not the complete instrument. This choice emerged as a mitigation measure to reduce the data collection burden and allow us to efficiently collect data on an aspect seldom reported in the literature. Future studies should explore the possibility of including complete validated tools, such as MTUAS [23] or the eHealth Literacy Scale [42]. We also need to consider that although the questionnaires were independently answered by the study participants, they were provided to them by their family physicians provided during an in-person visit, which may have influenced them to give desirable responses, namely those related to their general health status and asthma control.

Finally, most subjects included in this study were relatively young, had at least 7 years of education, were in the 3 higher quintiles of the socioeconomic deprivation index, had an overall good health status, and had good asthma control. Consequently, attempts to generalize these findings to other populations that do not share the same characteristics need to be cautious. Future studies should consider involving patients with specific needs (ie, lower education attainment, lower sociodemographic levels, poorer overall health status, or poorer asthma status control) and evaluate the replicability of these findings.

### Conclusions

Our results show a negative association with low literacy and the presence of depressive symptoms, highlighting the need for future research to explore the effect of disparities on app use, particularly among those with lower digital health literacy or mental health issues. Importantly, such studies should also assess participants’ general health-seeking behavior (ie, interest and participation in healthy lifestyle interventions in general) and the key constructs of their acceptance the unified theory of acceptance and use of technology) [43,44]. This theory was developed through a consolidation of the constructs of 8 previous models (theory of reasoned action, technology acceptance model, motivational model, theory of planned behavior, a combined theory of planned behavior/technology acceptance model, model of personal computer use, diffusion of innovations theory, and social cognitive theory), and includes four constructs: performance expectancy, effort expectancy, social influence, and facilitating conditions. Gender, age, experience, and voluntariness of use have been suggested to moderate the impact of the 4 key constructs on usage intention and behavior [43,44].

A better understanding of which patients with asthma are (and are not) using general health and fitness apps is key to design tailored mHealth interventions to improve sustained use by this specific group of patients. Additionally, this knowledge can also inform high-level delivery strategies to ensure that these solutions reach out comprehensive groups of patients with asthma, thus improving rather than entrenching health inequities within this population.

## Abbreviations

aOR: adjusted odds ratio
MTUAS: Media and Technology Usage and Attitudes Scale
VAS: Visual Analog Scale
